# Bioavailability and pharmacokinetic comparison of tanshinones between two formulations of *Salvia miltiorrhiza* in healthy volunteers

**DOI:** 10.1038/s41598-017-02747-4

**Published:** 2017-07-05

**Authors:** Lu Xing, Zhi-Rong Tan, Jin-Le Cheng, Wei-Hua Huang, Wei Zhang, Wen Deng, Chun-Su Yuan, Hong-Hao Zhou

**Affiliations:** 1 Department of Clinical Pharmacology, Xiangya Hospital, Central South University, Changsha, 410008 P.R. China; 2 Institute of Clinical Pharmacology, Central South University, Hunan Key Laboratory of Pharmacogenetics, Changsha, 410078 P.R. China; 3 Key Laboratory of Cell-broken Decoction Pieces Technology and Application of State Administration of Traditional Chinese Medicine, Zhongshan, 528437 P.R. China; 4 Tang Center for Herbal Medicine Research, The Pritzker School of Medicine, University of Chicago, Chicago, IL 60637 USA

## Abstract

*Salvia miltiorrhiza* (SM) is widely used to treat microcirculatory disturbance-related diseases; its lipophilic components play important roles in this application. Cryptotanshinone (CTS), tanshinone I (TSI) and tanshinone IIA (TSA) are the most widely-studied lipophilic ingredients, but low oral bioavailability limits their clinical application. It has been proven that micronization could improve the bioavailability of some drugs, so we’ve conducted this randomized study to investigate whether micronized granular powder (GP) of SM could improve the bioavailability of tanshinones compared with traditional decoction (TD). An oral dose of TD or GP of SM was administrated to subjects and blood samples were collected at predetermined time points. The plasma concentrations of tanshinones were detected by a validated method and pharmacokinetic parameters were calculated using a non-compartmental model. GP of SM resulted in a significant increase in mean maximum plasma concentration (*C*_*max*_), elimination half-life and area under concentration-time curve (AUC) of tanshinones, with the plasma AUC of CTS, TSI and TSA in GP 5–184, 4–619 and 5–130 times higher than TD. In addition, the individual variances of *C*_*max*_ and AUC were much lower after GP administration. Summarily, tanshinones in micronized GP of SM had higher oral bioavailability and lower individual variances, thus we speculate that it may indicate a better clinical efficacy and be a better choice than current treatments.

## Introduction

**Figure 1:**
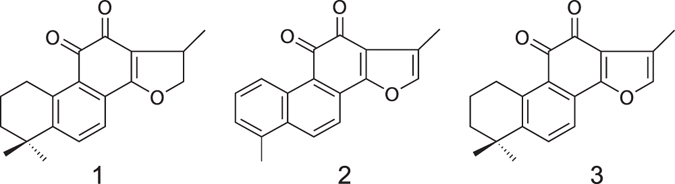
Structural formulas of cryptotanshinone (1), tanshinone I (2) and tanshinone IIA (3).

*Salvia miltiorrhiza* (SM, also known as Danshen, tanshen, Asian red sage), the dried root and rhizome of *Salvia miltiorrhiza* Bunge. in the family *Labiatae*, is widely used in China, Japan, the United States, and other European countries to treat various microcirculatory disturbance-related diseases, and has been for more than 2,000 years^1^. Cryptotanshinone (CTS), tanshinone I (TSI) and tanshinone IIA (TSA) (Fig. 1) are the major lipophilic compounds of SM with diterpenoidal quinone structure^2^ and sulfotanshinone sodium injection has already been used for the treatment of coronary heart disease, angina pectoris and myocardial infarction in clinical practices. These tanshinones have drawn attention for their possession of various pharmacological effects, including antibacterial^3^, antioxidant^4, 5^, anti-inflammatory^5, 6^, antineoplastic^7–13^, and cardio-cerebrovascular protection activities^14–16^.

However, the poor water solubility and low dissolution rate of tanshinones resulted in low oral bioavailability and had limited their clinical application^17^. To solve these problems, researchers have devised various methods loading tanshinones into micelles, liposomes, nanoparticles, microemulsions, cyclodextrin inclusions, solid dispersions, and so on^18–25^. These studies mainly focused on one or more phytochemicals extracted from SM rather than treating SM as a holistic herb. These preparations had only been studied *in vitro* or in animals; there is still a long way to go before these treatments yield health benefits in the clinic. It has already been established that herbal medicines have many components and are multi-targeted, and thus may fit well for the therapy of multi-gene related complex diseases^26–28^. Consequently, bioavailability enhancement technologies that take herbs as a whole are urgently needed for drug development.

Fortunately, micronization has been developed to increase solubility and dissolution through particle size reduction and surface area enhancement. Supercritical fluid technologies were applied in micronization for their advantages in producing solvent-free drug particles and homogeneous size distribution. The micronized technology was proven to be feasible for hesperidin and nimodipine^29, 30^ in improving bioavailability, while KW-2581 and telmisartan did not achieve the expected effect for decomposition^31, 32^. Hence, the application of micronization to herbal medicines with low solubility and bioavailability ingredients needs to be studied case by case.

Granule powder (GP) of SM was produced with micronization and increasingly used nowadays: crude SM were first crushed into micro-fine powder (particle size distribution, D_90_ < 45 *μ*m) with airflow crushing technology, and then made into dry granules without excipients^33^. The products preserved the active ingredients without heating or damaging them and slowed down the process of moisture absorption and decomposition.

SM and related formulas are widely prescribed in the clinic in China, and Compound Danshen Dripping Pills has entered the Phase III trials in the United States. However, pharmacokinetics of tanshinones have only been explored in animals, thus assessment of their pharmacokinetics and bioavailability in human is urgently needed. This study is designed to evaluate the effect of micronization on the pharmacokinetics of tanshinones and relative bioavailability of CTS, TSI and TSA in SM compared with traditional decoction (TD) dosage form. The results may provide insights into ways to use GP properly in clinical practice.

## Results

### Baseline Characteristics of Subjects and Adverse Events

Of 31 participants who gave their written consent, 24 were enrolled. All subjects completed both treatment periods and attended the visit at the end of study. The demographic characteristics of the participants are listed in Table 1. No statistically significant differences were observed in terms of age, body weight or body mass index between the two groups. No adverse events occurred during the clinical study.

**Table 1:** Summary of demographic characteristics of subjects.

Variables	Treatment sequence		All
	TD/GP	GP/TD	
N (male)	6	6	12
N (female)	6	6	12
Age (years)	25.3 ± 2.3	23.3 ± 2.6	24.3 ± 2.6
Height (cm)	164.3 ± 7.1	167.5 ± 8.4	165.9 ± 7.8
Body weight (kg)	58.0 ± 7.5	60.4 ± 8.5	59.2 ± 7.9
BMI (kg/*m* ^2^)	21.4 ± 1.5	21.5 ± 1.9	21.5 ± 1.7

Data are expressed as mean ± standard deviation. N number, BMI body mass index, TD traditional decoction of *Salvia miltiorrhiza*, GP granular powder of *Salvia miltiorrhiza*.

### Determination of CTS, TSI and TSA Concentrations *in vitro*

Concentrations of tanshinones before administration to subjects were detected and all tanshinones were higher in GP preparations than TD. Compared with TSA and TSI, the content of CTS was highest regardless of in GP or TD, with the concentration of 10.939 *μ*g/mL in the supernatant of GP and 5.257 μg/mL in TD. Interestingly, TSA had lower content than TSI in TD (0.681 and 0.839 *μ*g/mL respectively), but was more than twice of TSI in GP supernatant (2.097 and 0.919 *μ*g/mL respectively). The ratios of tanshinones in GP/TD were 2.02 for CTS, 1.10 for TSI and 3.08 for TSA, respectively.

### Pharmacokinetics

**Figure 2:**
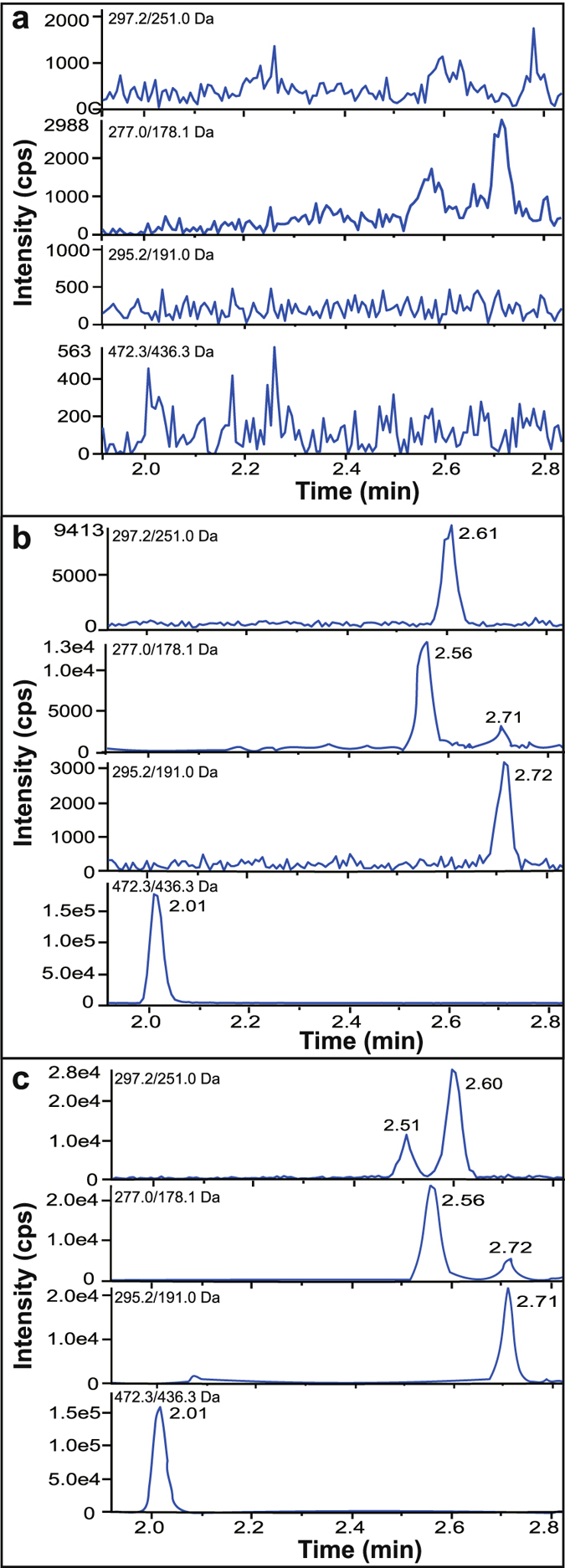
Representative multiple reaction monitoring (MRM) chromatograms of cryptotanshinone (CTS), tanshinone I (TSI), tanshinone IIA (TSA) and terfenadine (IS) in blank plasma (**a**), plasma spiked with tanshinones at LLOQ and IS (**b**), and plasma sample collected at 8 h after dosage (**c**).

**Figure 3:**
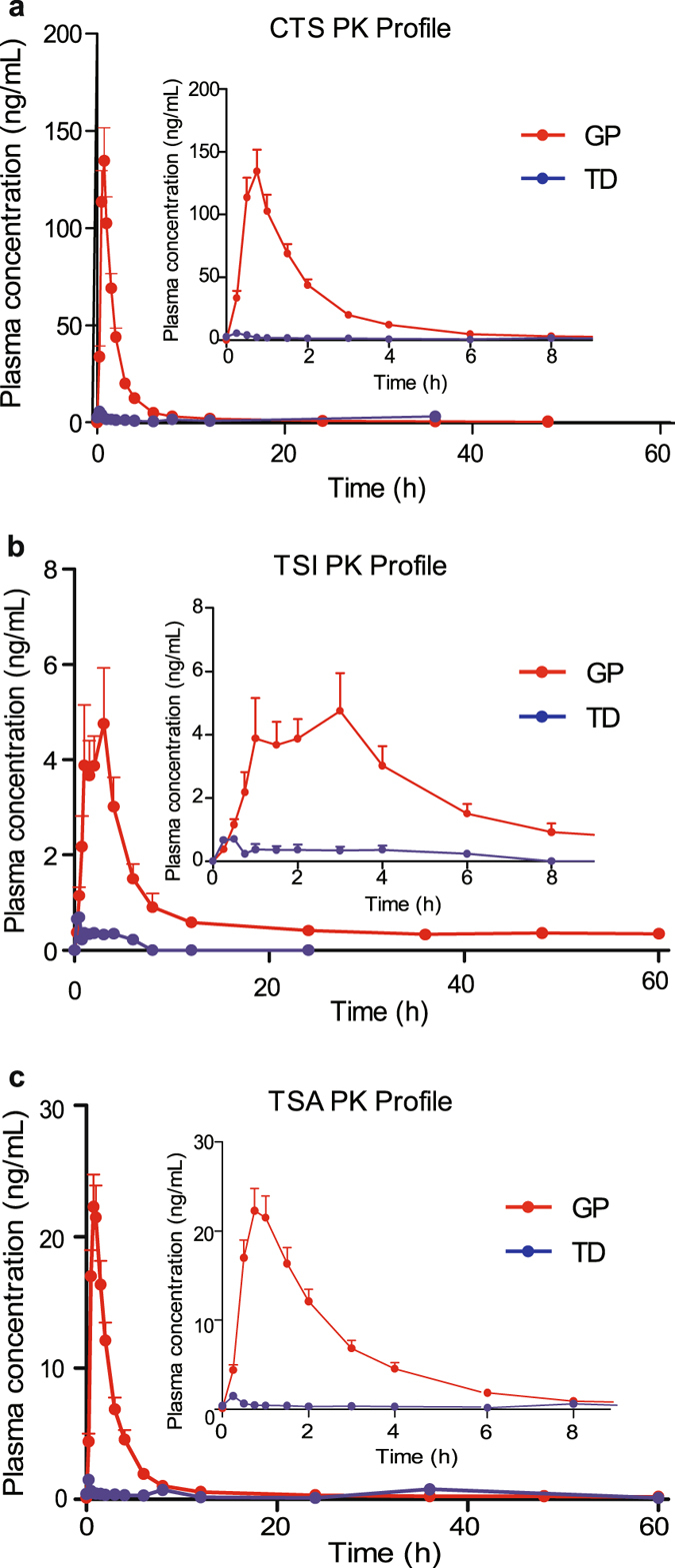
Arithmetic mean (±standard error) plasma concentration–time profiles of cryptotanshinone (**a**), tanshinone I (**b**) and tanshinone IIA (**c**) after administration of *Salvia miltiorrhiza* granule powder (GP) and traditional decoction (TD).

Specificity, accuracy, precision, recovery and matrix effects all satisfied the guidelines. The linear ranges were 0.4–200 ng/mL for CTS, 0.2–100 ng/mL for TSI and 0.1–50 ng/mL for TSA, respectively. Representative chromatograms of blank human plasma, spiked lower limit of quantification (LLOQ) and plasma sample collected after dosage are presented in Fig. 2. No endogenous peaks were found to interfere at the retention time of any of the analytes. Figure 3 depicts the mean plasma concentration-time profiles of CTS, TSI and TSA, which were characterized by a tremendous increase in plasma concentrations and overall exposure after administration of GP compared with TD. The pharmacokinetic parameters calculated for CTS, TSI and TSA after administration of TD and GP of SM are presented in Table 2.

**Table 2:** The pharmacokinetic parameters and relative bioavailability of tanshinones in 24 subjects after administration of GP and TD of SM.

PK parameters	Cryptotanshinone			Tanshinone I			Tanshinone IIA		
	TD	GP	*p* value	TD	GP	*p* value	TD	GP	*p* value
*C*_*max*_ (ng/mL)^*a*^	6.37 ± 6.45	146.72 ± 87.61	0.000	0.43 ± 0.97	6.57 ± 7.09	0.000	1.75 ± 4.56	25.76 ± 12.89	0.000
*T*_*max*_ (h)	0.39 ± 0.13	0.80 ± 0.38	0.000	2.35 ± 2.09	1.85 ± 1.17	0.620	0.53 ± 0.30	0.91 ± 0.32	0.000
*t*_1/2_ (h)	2.64 ± 3.95	5.22 ± 3.22	0.000	2.45 ± 0.33	9.42 ± 11.28	0.568	4.50 ± 6.77	14.80 ± 13.44	0.000
AUC_0−*t*_ (ng h/mL)^*a*^	9.05 ± 11.75	251.70 ± 124.85	0.000	0.60 ± 0.96	30.48 ± 29.19	0.000	3.06 ± 4.11	69.72 ± 40.79	0.000
AUC_0−∞_ (ng h/mL)^*a*^	11.53 ± 12.69	256.00 ± 126.24	0.000	0.76 ± 1.20	33.80 ± 31.58	0.000	3.69 ± 4.20	73.42 ± 44.12	0.000
BA (%)	4,456.8 ± 3,709.8			1,2474.8 ± 2,0057.6			4690.3 ± 3416.7		
normalized BA (%)	2,141.9 ± 1,782.9			11,388.9 ± 1,8311.6			1,523.7 ± 1,110.0		

Data are expressed as mean ± standard deviation, n = 24. ^*a*^ *p* values were analyzed after logarithmic transformation. SM *Salvia miltiorrhiza*, TD traditional decoction of *Salvia miltiorrhiza*, GP granular powder of *Salvia miltiorrhiza, C*_*max*_ peak plasma concentration*, T*_*max*_ time to reach the *C*_*max*_, *t*_1/2_ elimination half-life, *AUC*_0−*t*_ area under the plasma concentration–time curve from time zero to time t, *AUC*_0−∞_ area under the plasma concentration–time curve extrapolated to infinity, BA bioavailability.

**Figure 4:**
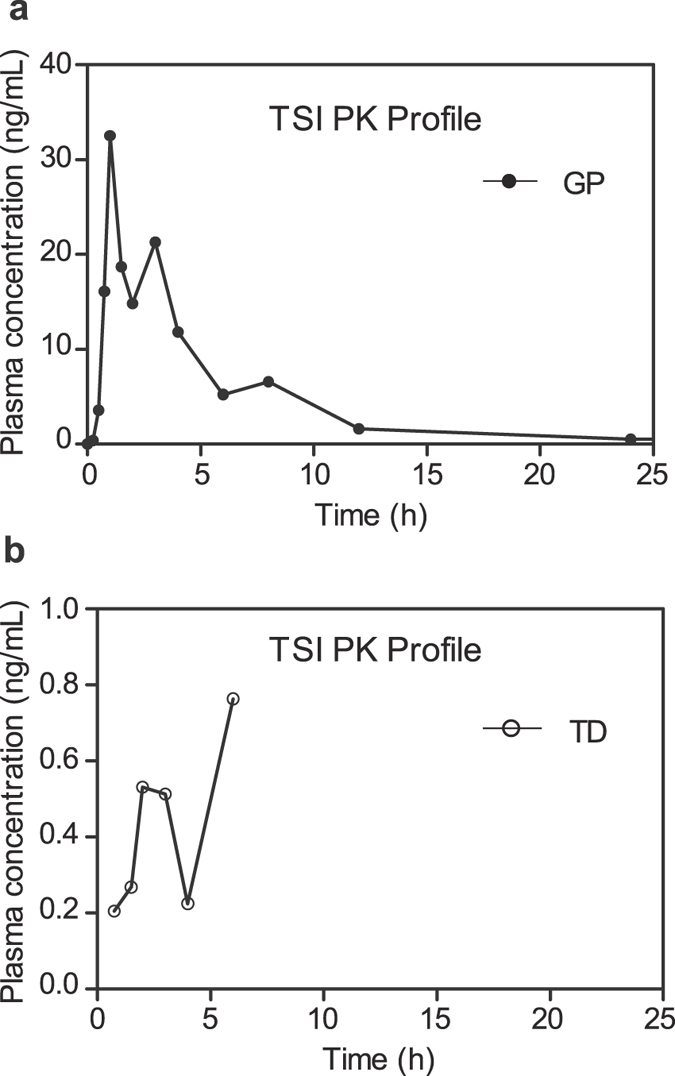
Plasma concentration-time profiles for tanshinone I in subject 15 after oral administration of *Salvia miltiorrhiza* (**a**) granule powder (GP) and (**b**) traditional decoction (TD).

CTS exhibited a maximum plasma concentration (*C*_*max*_) of 6.37 ng/mL at about 0.39 h after TD administration, and the plasma concentration decreased quickly thereafter with an elimination half-life (*t*_1/2_) of about 2.64 h. While for GP, *C*_*max*_ of CTS reached to 146.72 ng/mL (23 times of TD), the time of *C*_*max*_ occurrence (*T*_*max*_) shifted to approximately 0.80 h and *t*_1/2_ increased to 5.22 h (2-fold of TD). As for TSI, the plasma concentration in most subjects was under LLOQ after administration of TD and it was completely undetectable in 11 subjects. While after administration of GP, the plasma concentration-time profiles of TSI in most subjects (20/24) exhibited two or three distinct peak concentration values (typical profiles are shown in Fig. 4), resulting in large variances in *C*_*max*_, *T*_*max*_ and *t*_1/2_. *C*_*max*_ of TSI was 0.21–4.80 ng/mL and 1.84–32.5 ng/mL after administration of TD and GP respectively, the *T*_*max*_ ranged from 0.5 h to 6 h and the *t*_1/2_ varied from 0.8 h to 39 h in GP group. TSA achieved its *C*_*max*_ (1.75 ng/mL) at about 0.53 h after TD dosing, with a calculated *t*_1/2_ of nearly 4.50 h, while with GP administration, its *T*_*max*_ shifted to approximately 0.91 h with a *C*_*max*_ of 25.76 ng/mL (about 14.7 times of TD) and the mean *t*_1/2_ increased to probably 14.80 h.

**Figure 5:**
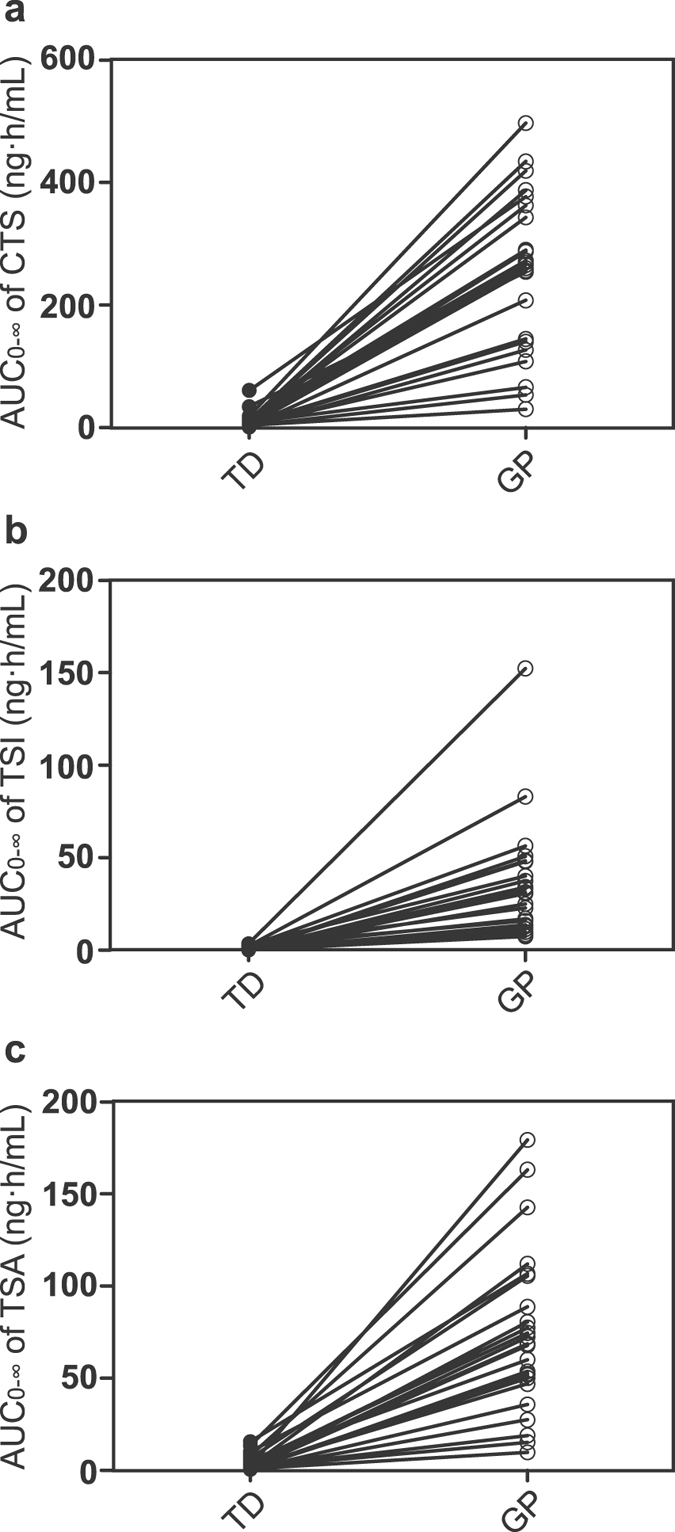
Comparison of individual area under the plasma concentration–time curve (AUC) values of cryptotanshinone (**a**), tanshinone I (**b**) and tanshinone IIA (**c**) obtained after granule powder (GP) and traditional decoction (TD) administration of *Salvia miltiorrhiza*. Each line in the plots represents an individual study participant. Plots with filled circles represent the AUC_0−∞_ of traditional decoction (TD), and those with open circles represent the AUC_0−∞_ of granule powder (GP).

Relative bioavailability increased 43.6, 123.7 and 45.9 times for CTS, TSI and TSA, respectively. Even after dose normalization, the elevated bioavailability was still noticeable (Table 2). However, the area under the plasma concentration-time curve to time infinity (AUC_0−∞_) of tanshinones varied considerably between individuals (Fig. 5). Similarly, the *C*_*max*_ of tanshinones showed a wide inter-subject variability, especially after TD administration (Table 3). The overall coefficient of variation (CV) of CTS in TD and GP was 101.3% and 59.7%, respectively. The CV of CTS ranged from 44.1% to 62.4% in male subjects, and from 90.2% to 59.5% in female subjects for TD and GP respectively. The CV of TSI in TD and GP was 127.8% and 102.3% in male subjects, while it became larger in female subjects (206.5% and 114.8% for TD and GP respectively) and CV of TSI was 226.4% and 108.0% in TD and GP on the whole. Individual variance of TSA in TD and GP were almost unchanged from 55.1% to 53.9% in male subjects but reduced considerably from 73.5% to 48.3% in female subjects, resulting in a declining overall CV from 72.8% to 50.1%.

**Table 3:** Individual variance of *C*_*max*_ of tanshinones after administration of GP and TD of SM.

Tanshinones	Gender	TD		GP	
		*C*_*max*_ (ng/mL)	CV (%)	*C*_*max*_ (ng/mL)	CV (%)
Cryptotanshinone	Male	3.60 (1.26–7.01)	44.1	141.40 (26.00–301.00)	62.4
	Female	9.13 (2.29–26.70)	90.2	152.00 (14.40–291.00)	59.5
	All	6.37 (1.26–26.70)	101.3	146.70 (14.40–301)	59.7
Tanshinone I	Male	0.21 (0.00–0.70)	127.8	6.57 (1.84–24.10)	102.3
	Female	0.65 (0.00–4.80)	206.5	7.09 (2.12–32.50)	114.8
	All	0.43 (0.00–4.80)	226.4	6.57 (1.84–32.50)	108.0
Tanshinone IIA	Male	0.64 (0.32–1.41)	55.1	24.70 (5.12–48.30)	53.9
	Female^*a*^	1.02 (0.34–2.52)	73.5	26.80 (3.76–48.30)	48.3
	All^*a*^	0.82 (0.32–2.52)	72.8	25.80 (3.76–48.30)	50.1

Data are expressed as mean (range). ^*a*^Data of subject 13 was excluded (outlier calculated by F-test). SM *Salvia miltiorrhiza*, TD traditional decoction of *Salvia miltiorrhiza*, GP granular powder of *Salvia miltiorrhiza*, *C*_*max*_ peak plasma concentration, CV coefficient of variation.

## Discussion

Previous studies about SM in human subjects mainly focused on phenolic constituents^34, 35^, this study is the first work investigating pharmacokinetics and relative bioavailability of CTS, TSI, TSA between TD and GP of SM in human. Tanshinones were found emerging rapidly in plasma, with half-life ranging 2–5 h in TD, indicating that they were absorbed quickly with a slower elimination half time, which was consistent with the results in rats and rabbits^36^. The same tendency was found after GP administration, although the *T*_*max*_ and *t*_1/2_ were prolonged. According to the study of Song *et al*., *T*_*max*_ and *t*_1/2_ of CTS and TSA were 0.58 ± 0.14 h and 3.81 ± 1.01 h, 0.64 ± 0.07 h and 5.12 ± 0.08 h, after intra-gavage of 23.3 mg/kg ethanol extracts of SM in Sprague–Dawley rats^37^. A pharmacokinetic study of Sodium Tanshinone IIA Sulfonate (STS) in Chinese healthy male volunteers showed that *t*_1/2_ was only 1 ± 0.8 h after receiving 40 mg STS administered as intravenous infusion^38^.

TSA exhibited lower content than TSI in TD *in vitro*, but plasma concentration of TSA was much higher than TSI after administration of TD. This contradiction could be explained by metabolization from CTS to TSA *in vivo*, according to the studies of Xue M, Song M and Hao H^39–41^. In addition, the absence of TSI in TD group might be owing to its lower content and extensive first pass effect. Data in rats showed that CTS and its metabolites were mainly eliminated from bile and multiple peaks were observed in the plasma concentration–time profiles^42^. Data in this study indicated multiple peaks in CTS and TSA after TD administration and in TSI after GP administration, whereas no obvious multiple peaks were observed in CTS or TSA after GP administration. The mechanism implied in multi-peak phenomenon of TSI (with structure similar to CTS) might be enterohepatic recycling process and high tissue concentrations^42, 43^. The absence of multiple peaks in CTS and TSA in most subjects might be due to the coverage of high plasma concentration.

Although the concentration of tanshinones in GP was only 1–3 times of TD *in vitro*, the plasma AUC_0−*t*_ of CTS, TSI and TSA in GP was 6–185, 5–620 and 6–131 times of TD. While micelles encapsulated with TSA improved the bioavailability of TSA only by 3–4 times^25^, and AUC_0−*t*_ of TSA ternary solid dispersion pellets in male New Zealand rabbits after orally administered was 0.76 times more than that of binary solid dispersion pellets, 2.87 times more than that of physical mixtures and 5.40 times more than that of TSA^21^. When CTS was dosed at 100 mg/kg in rats, the intraperitoneal bioavailability was 4 times more than that of orally administrated^44^. AUC_0−*t*_ of STS from ten subjects received 40 mg dose (about 100 times of TSA in GP) of STS injection was 742.0 ± 150.3 ngh/mL^38^, only 10 times of AUC_0−*t*_ of TSA (69.72 ± 40.79 ngh/mL) in GP. The significantly high bioavailability of tanshinones in GP could be due to any of the following reasons. First, it may be attributable to different proportions among the components in GP and TD. Studies had indicated that coexisting tanshinones in SM could improve the *C*_*max*_ and AUC for CTS and TSA^37^, and significant increase for TSA was observed in high dose groups when combined with polyphenolic extracts of SM in rats^45^. Second, high binding power of salvianolic acid B and TSA to plasma protein^46–48^ also contributed to the phenomenon. Moreover, TSA is a substrate and reversing agent for P-glycoprotein (P-gp) and coexisted components of SM could enhance intestinal absorption of CTS via inhibition of the intestinal P-gp^49, 50^.

This study indicated that tanshinones had large inter-individual variation in *C*_*max*_ and AUC. Female volunteers had higher *C*_*max*_ than male volunteers, although statistically significance (*p* = 0.000) was only found in CTS after administration of TD (Table 3). This may be associated with genetic polymorphisms of enzymes involved in absorption, distribution, metabolism and excretion of tanshinones^51^, but further investigations are needed to better understand mechanisms underlying the phenomenon. The variations were found to be larger in TD for CTS, TSI and TSA on the whole, revealing that GP of SM had more stable qualities and micronization probably provided a new way to the quantify control of herbal medicines. In addition, the preparation of GP is more convenient, we thus suggest that, for achieving clinical efficacy, the GP dosage form of SM is a better choice, although the dosage should be reduced and dosing intervals prolonged.

Micronization of herbal medicines increased specific surface area and enhanced dissolution rate^52^, thus theoretically improved the bioavailability *in vivo*. Here we demonstrated that it was true for the lipophilic components in SM, and micronization would be of great benefit for herbal medicines with similar properties. For example, poor oral bioavailability of curcumin was due to its limited intestinal uptake and rapid metabolism, while micronized powder and liquid micellar formulation of curcumin could significantly improve its oral bioavailability^53^. This technology could also be used for the protection of herb germplasm, especially for scare and precious herbs. On the other hand, the dissolution of toxic and active ingredients can be increased at the same time, and increased plasma concentrations are often associated with increased adverse reactions. However, no adverse reactions were observed in our study, which may be due to the wide safety of SM, but the toxicities need further observation in clinical applications.

Despite the inspiring results, our study has some limitations. According to guidelines of Chinese State Food and Drug Administration (CFDA) on the pharmacokinetic study, 8–12 subjects are required for each group. Therefore, twenty-four subjects were enrolled according to the study protocol. However, differences among individuals indicated that the sample size needed to be enlarged. In addition, only a single dose of GP was tested in the study. TD of SM has shown its efficacy and safety for thousands of years, so it was choosed as a reference for the relative bioavailability study. While coarse powder of SM is also applied in clinical practice, the inclusion of coarse powder as another reference for the study will make the results more convincing. Chinese Pharmacopoeia’s recommended dose for SM in decoction form is 10–15 g, and higher doses up to 30–60 g can be used according to English-Chinese Rare Chinese Materia Medica. However, our preliminary study (included 4 male subjects) with 10 g SM resulted in mean *C*_*max*_ of TSA at about 1 ng/mL in TD. Furthermore, SM has very low toxicity, the acute toxicity (LD_50_) is 25.8 g/kg in mice when a water-soluble SM extract was administered orally, and an oral dose of 2.5 g/kg SM extract for 90 days was found to be nontoxic to rats^54^. Normally an adult weighs 60 kg, so 20 g SM (0.33 g/kg, much lower than LD_50_) employed in this study was extremely safe. Guo *et al*. had observed about 2 fold increase in low and medium dose groups and 14 fold significant increase in high dose group for TSA, which was due to competitive distribution and metabolism of other ingredients^45^. These results implied that tanshinones exhibited non-linear plasma pharmacokinetic properties after SM administration, which has been revealed by Dai H and Yu XY^49, 50^. Therefore, assessment of one or more groups taking less GP should be carried out for a full understanding of the relative bioavailability between GP and TD.

## Conclusions

In conclusion, our research indicated that GP of SM had greater *C*_*max*_ and AUC, longer *T*_*max*_ and *t*_1/2_ than TD, resulting in higher bioavailability of tanshinones than TD. An increased plasma exposure of tanshinones after GP administration might affect the pharmacological effects and safety of SM. The dosage of GP should be reduced when used in the clinic by a physician. The next step is to develop a reasonable dosage regimen for GP of SM. Additionally, the formulation was more homogeneous and the preparation is more convenient after micronization, this technology could improve the quality control and promote the modernization of herbal medicines mainly containing lipophilic components with wide safety.

## Methods

### Subjects Selection

Subjects were screened between day −14 and day −1 of period 1 using predefined inclusion and exclusion criteria to select those who did not have conditions that would present undue safety risks or interfere with the absorption or metabolism of study drugs. Inclusion criteria were as follows: subjects of Han Chinese origin, aged 18–40 years, body mass index 18–24 kg/m^2^, and healthy by medical history, laboratory test results, electrocardiogram, vital signs and physical examination. Women were required to have a negative pregnancy test. Any medications, alcohol, tobacco, grapefruit juice, coffee, green tea and nutritional supplements were prohibited for 2 weeks prior to and during the study.

Subjects were provided with written, informed consent prior to study participation. The study was reviewed and approved by Independent Ethics Committee Board of the Institute of Clinical Pharmacology, Central South University (No. CTXY-150011-4, 29th June 2016). The registration number (ChiCTR-OIC-16008757), date of registration (1st July 2016) and the trial protocol were validated on Chinese Clinical Trial Register. The study was carried out in Hunan Cancer Hospital according to the protocol, the World Medical Association Declaration of Helsinki, and the WHO Guideline for Good Clinical Practice.

### Preparations of TD and GP

GP and crude herb of SM (lot number: 20150803) were provided by Zhongshan Zhongzhi Pharmaceutical Group Co., Ltd. (Guangdong, China). The details of the preparations were as follows.

**TD** Traditional crude SM (260 g) was first soaked in 2,500 mL cold water for 30 minutes, then decocted in an electrical casserole with big fire (180–200 °C) before boiling and maintained a slight boiling state (120–150 °C) for 30 minutes. After filtrating, dregs of crude SM were decocted for another 30 minutes with 1,000 mL water. The filtrate was combined and adjusted to 2,600 mL, then exactly separated into 13 portions, and stored in 4 °C refrigerator. TD of SM was warmed to an appropriate temperature at about 45 °C (equal to that of GP) for the subjects to drink.

**GP** GP of SM (20 g) was added to 100 mL warm water (60 °C) and stirred with a cooking machine for one minute. The plastic mixing cup was immediately washed by 66 mL warm water and all the suspensions were poured into a glass cup, then administrated to subjects after 30 minutes. Another 20 mL warm water washing the glass was also administered to subjects, making sure the total volume of administration is 200 mL, equal to that of TD.

Concentrations of tanshinones in TD and GP supernatant were detected by HPLC-UV after centrifugation (13,000 rpm for 10 minutes).

### Study Design

This was a randomized, open-label, single-center, single-dose, 2-period crossover study to assess the pharmacokinetics of CTS, TSI and TSA in healthy Chinese volunteers. Twenty-four subjects (12 males and 12 females) who satisfied the selection criteria were equally randomized to 2 groups (random numbers were generated by Drug and Statistic software, version 3.2.2 after sorting by BMI and gender) and received a single oral dose of TD or GP (both contained 20 g crude SM) on study days 1 and 8 after fasted overnight. Blood samples for the pharmacokinetic analysis were drawn at pre-dose and at 0.25, 0.5, 0.75, 1, 1.5, 2, 3, 4, 6, 8, 12, 24, 36, 48 and 60 h after the dose. A 5 mL aliquot of blood was collected into a glass evacuated tube containing ethylenediaminetetraacetic acid disodium salt (EDTA-Na_2_). Plasma was separated by centrifugation (3,000 rpm for 10 min at 4 °C) within 30 min of collection and stored at −40 °C until analysis.

### Safety Evaluation

The entire study was conducted under medical supervision and all side effects were collected. The safety assessment included clinical evaluation, scheduled laboratory testing on day 11, and physical examinations (blood pressure, pulse, body temperature, and respiration) monitored at −12, 0, 4, 8, 12 and 24 h after drug administration.

### Pharmacokinetic Assessments

Measurement of plasma concentrations of CTS, TSI and TSA was performed after sample preparation by high pressure liquid chromatography-tandem mass spectrometry (HPLC-MS/MS) on API5500 QTRAP triple quadrupole mass spectrometer (Applied Biosystem SCIEX, Foster City, USA).

In detail, plasma samples (50 *μ*L) were spiked with 30 *μ*L acetonitrile–methanol (1:1, v/v, containing 5 ng/mL internal standard terfenadine), and underwent liquid–liquid extraction with 600 *μ*L methyl tertiary butyl ether (MTBE). After evaporation of the organic phase, the residues were reconstituted with 150 *μ*L acetonitrile–water (6:4, v/v, containing 0.1% formic acid) and 2 *μ*L of each sample was injected into the HPLC-MS/MS system. Since tanshinones are photosensitive, plasma samples were protected from ambient light during sample preparation.

LC was carried out on a Phenomenex Kinetex C_18_ column (50 × 2.1 mm, 5 *μ*m) with a LC-20AD pump equipment (Shimadzu, Kyoto, Japan). Gradient elution (0–0.5 min 10% B, 0.5–2 min 10–95% B, 2–3 min 95% B, 3–3.01 min 95–10% B, 3.01–4 min 10% B) delivered at 0.5 mL/min was applied with mobile phase A (0.1% formic acid in water) and mobile phase B (0.1% formic acid in acetonitrile). Subsequently, MS/MS analysis was performed by electrospray ionization (ESI) in the positive ion mode. Mass transitions from m/z 297.2 to 251.0, 277.0 to 178.1, 295.2 to 191.0 and 472.3 to 436.3 were monitored for CTS, TSI, TSA and the internal standard terfenadine respectively.

Method validation was performed in accordance with the currently accepted US Food and Drug Administration (FDA) bioanalytical method validation guide (Bioanalytical Method Validation, 2001).

### Pharmacokinetic and Statistical Analysis

Pharmacokinetic parameters were estimated using non-compartmental methods (Drug and Statistic software, version 3.2.2, Clinical Drug Evaluation Center, Wannan medical College, Anhui, China). *C*_*max*_ and *T*_*max*_ were obtained directly from the observed data. *C*_*t*_ was defined as the last observed quantifiable concentration after the dosage. The elimination rate constant (*k*_*e*_) was calculated by log-linear regression of concentrations observed during the terminal phase of elimination. The *t*_1/2_ was calculated as ln 2/*k*_e_. The area under the plasma concentration-time curve to the last measurable plasma concentration (AUC_0−*t*_) was calculated using the linear trapezoidal method. AUC_0−∞_ was calculated as AUC_0−*t*_ + *C*_*t*_/*k*_e_. Relative bioavailability was calculated by AUC_0−*t*,GP_/AUC_0−*t*,*TD*_ × 100%. Statistical analysis was performed using SPSS software. The differences between *C*_*max*_ and AUC were determined with independent samples t test, and the Mann–Whitney test was applied to *t*_1/2_ and *T*_*max*_. The CVs(%) were calculated as mean/SD (standard deviation) × 100. A *p* value of <0.05 was considered significant.
